# Sagittal spinopelvic parameters in 2-level lumbar degenerative spondylolisthesis: A retrospective study

**DOI:** 10.1097/MD.0000000000005417

**Published:** 2016-12-16

**Authors:** Tao Wang, Hui Wang, Huan Liu, Lei Ma, Feng-Yu Liu, Wen-Yuan Ding

**Affiliations:** Department of Spinal Surgery, The Third Hospital of Hebei Medical University, Shijiazhuang, China.

**Keywords:** 2-level, degenerative spondylolisthesis, sagittal spinopelvic alignments, single-level

## Abstract

The purpose of our study is to evaluate sagittal parameters in 2-level lumbar degenerative spondylolisthesis (DS) (TLDS).

A total of 15 patients with TLDS, 40 patients with single-level DS (SLDS), and 30 normal volunteers as control were included in our study. All subjects performed on full spine X-ray. Two categorized data were analyzed: patient characteristics—age, sex, body mass index, radiographic parameters-pelvic incidence (PI), pelvic tilt (PT), lumbar lordosis (LL), sacral slope (SS), PI–LL, Cobb between the fifth thoracic vertebral and 12th thoracic vertebral (T5–T12), sagittal vertical axis (SVA) Cobb angle of spondylolisthesis level (CSL), ratio of PT to SS (PT/SS), CSL/LL, variation trend of SS over PI, and LL over PI.

The PI (73.1° vs 52.9°), SS (50.8° vs 32.2°), LL (53.1° vs 46.9°), SVA (66.1 vs 22.0 mm), PI–LL (20.0° vs 6.0°), and CSL (23.6° vs 20.0°) in TLDS were significantly larger than these in SLDS. The PI (73.1° vs 40.6°), PT (22.3° vs 17.1°), SS (50.8° vs 23.5°), LL (53.1° vs 32.5°), PI–LL (20.0° vs 8.1°), and SVA (66.1 vs 17.0 mm) in TLDS were significantly larger than those in the normal group (NG). The PI (52.9° vs 40.6°), PT (21.0° vs 17.1°), SS (32.2° vs 23.5°), LL (46.9° vs 32.5°), and SVA (22.0 vs 17.0 mm) in SLDS were significantly higher than those in NG. However, PT/SS (44.0%), LL over PI (y = 0.39x + 24.25), SS over PI (y = 10.79 + 0.55x) were lower in TLDS than these in SLDS (63.8%, y = 0.41x + 25, y = 0.65x − 2.09, respectively), and the similar tend between SLDS and NG (74.0%, y = 0.49x + 13.09, y = 0.67x − 3.9, respectively).

Our results showed that 2-level lumbar DS, which was caused by multiple-factors, has a severe sagittal imbalance, but single-level has not any. When we plan for surgical selection for 2-level lumbar DS, global sagittal balance must be considered.

## 1 Introduction

Spondylolisthesis, including degenerative spondylolisthesis (DS) and isthmic spondylolisthesis (IS), is a common degenerative spinal disease, described as a condition that compared to lower vertebral body, upper vertebral body shift forward with an intact neural arch^[1–3]^. Two-level DS (TLDS) is 2 consecutive vertebral body shift forward with an intact neural arch, similarly single-level DS (SLDS) is only 1 vertebral body shift forward. Garet et al^[4]^ reported that incidence of spondylolysis ranges from approximately 6% to 11.5% in the general population. DS is an important type of spondylolisthesis and lumbar degenerative disorder leading to low back pain, disability, and neurological deficit.^[3–5]^ Previously, we only pay attention to regional problem in treatment of DS, as a neural decompression and obtaining a bony fusion. Along with development of spine, sagittal alignment is considered as an important key in surgical treatment for spinal degenerative diseases.^[2–10]^

Duval-Beaupère et al^[11]^ described pelvic incidence (PI), a fundamental anatomical parameter, which is unique for each individual and does not depend on the position or spatial orientation of the pelvis. Also Duval-Beaupère^[12]^ was the first to put forward the importance of pelvic indexes and their relationship (PI = PT + SS). PI is closely related to sacral slope (SS) and pelvic tilt (PT), 2 position-dependent variables that determine pelvic orientation in the sagittal plane. A series of studies reported spino-pelvic parameters were important factors for degeneration spinal diseases, such as lumbar disc degeneration or lumbar disc herniation (LDH) and DS^[13–18]^. Rajnics and Endo^[15,16]^ reported a common pattern of spinopelvic sagittal alignment in patients with LDH, which is characterized by lower SS, lower lumbar lordosis (LL), and anterior translation of the C7 plumb line. Sanderson and Fraser^[19]^ and Matsunaga et al^[20]^ reported that pregnancy and joint laxity may lead to DS. Sato et al^[21]^ demonstrated that the configuration of the laminas and sagittal facet joints were contributing factors for DS. Funao et al^[22]^ showed that greater PI may be predisposing factors to the development and the progression of vertebral slip due to DS.

Relatively few published studies discussed the characteristics of sagittal alignment in patients with DS, especially 2-level, which is rare in clinic. The purpose of our work is to explore the sagittal parameters for TLDS and compare the difference between TLDS and SLDS in sagittal parameters, providing some suggestions for surgical treatment in 2-level lumbar DS in clinic. In addition, we try to explore the etiology of TLDS.

## 2 Materials and methods

### 2.1 Subjects

The study was approved by the Institutional Review Board of the Third Hospital of HeBei Medical University before data collection and analysis. The inclusion criteria included lumbar spondylolisthesis with bilateral intact neural arches, belong to low grade (Meyerding I–II) and subjects adopt the standard position which is hands on clavicles. The exclusion criteria consisted of patients with IS, have history of any spinal surgery (including simple lumbar discectomy), have spinal deformities (including scoliosis, irregular endplate, sacralization, or lumbarization), have spinal trauma or tumors, absence of arthropathy in the lower limbs, and belong to high grade (Meyerding III–IV). A total of 15 patients with TLDS (8 subjects with L3–L4 spondylolisthesis and 7 subjects with L4–L5 spondylolisthesis) and 40 patients with SLDS (18 subjects with L4 spondylolisthesis and 22 subjects with L5 spondylolisthesis) were included in our study from the Third Hospital of HeBei Medical University in this study, from January 2013 to September 2015. Besides, 30 volunteers without symptom took part in our study from January 2013 to September 2015.

### 2.2 Radiological assessment

Lateral full spine X-ray for each subject was measured to assess sagittal alignment of the spine. The subject assumed a comfortable standing position with the knees fully extended and upper limbs raised horizontally forward at 45° of flexion at the shoulder resting on 2 arm supports. The following variables were measured as follows—PI: defined as the angle between the perpendicular to the upper sacral endplate at its midpoint and the line connecting this point to the femoral head. PT: Angle between vertical line and line joining hip axisa to center of superior endplate of S1. SS: Angle between superior endplate of S1 and horizontal line. LL: Segmental angle of superior endplate of L1 and superior endplate of S1. Cobb from the fifth thoracic vertebral to 12th thoracic vertebral (T5–T12) (TK): Cobb angle of superior endplate of T1 and inferior endplate of T12. PI–LL: Angle equals PI minus LL. Sagittal vertical axis (SVA): The horizontal distance between the posterior corner of the sacrum and the C7 plumb line. A positive value was defined when the sacral posterior corner landed in front of the C7 plumb line. Segmental Cobb angle of spondylolisthesis level (CSL): Segmental Cobb angle of superior endplate of spondylolisthesis vertebral and superior endplate of lower spondylolisthesis vertebral. CSL/LL: Divided Cobb of spondylolisthesis level by LL. LL over PI: Linear correlation between LL and PI. SS over PI: Linear correlation between SS and PI. Body mass index (BMI): Divided weight (kg) by the square of height (m).

### 2.3 Statistical analysis

The methods were carried out in accordance with the approved guidelines. Two authors identified and collected all the data of patients according to inclusion criteria and exclusion criteria. In addition, 2 authors (TW and HW) were responsible for data analyses. All measurement data are presented as the mean ± standard deviation when data satisfied criteria for normality with *P* > 0.05. Otherwise, it should be presented as median (interquartile range). These PI, PT, LL, SS, PT/SS, T5-T1, SVA, CSL, age, BMI, and CSL/LL satisfied criteria for normality and homogeneity of variance; statistical analysis between groups was performed using independent samples *t* test. In addition, count data, like sex (male/female), chi-square test was used for data analysis. The Kolmogorov–Smirnoff test was used to verify the normal data distribution. LL over PI and SS over PI were tested by linear regression model. Statistical significance levels were considered to be *P* < 0.05. All statistical analyses were carried out using SPSS, version 21.0 (SPSS Inc., Chicago, IL).

## 3 Results

**Figure 1:**
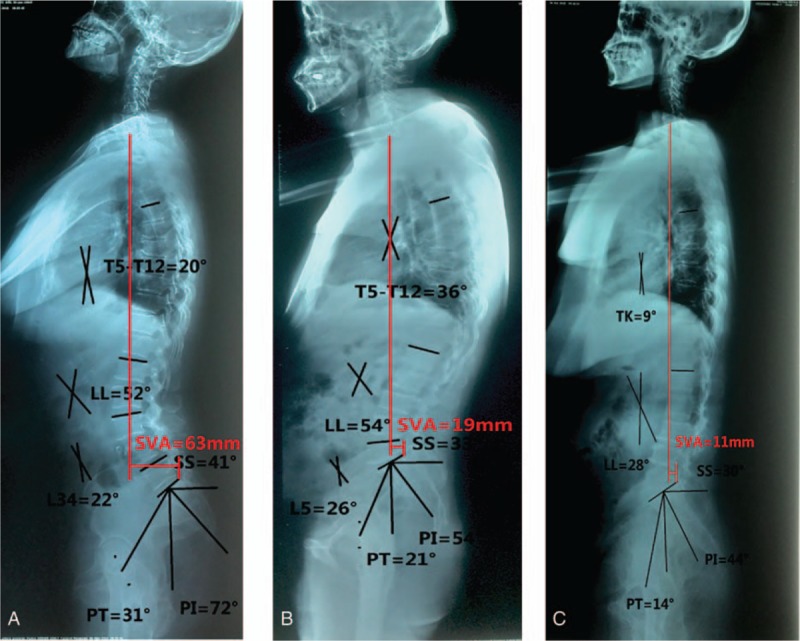
(A) A male, 47-year old. The lateral full spine X-ray shows L3 to L4 spondylolisthesis. PI = 72°, PT = 31°, SS = 41°, LL = 52°, T5 to 12 = 20°, CSL = 22°. (B) A male, 49-year old. A lateral full spine X-ray shows L5 spondylolisthesis. PI = 54°, PT = 21°, SS = 33°, LL = 54°, T5 to 12 = 36°, CSL = 26°. (C) A male, 48-year old. A lateral full spine X-ray shows no lumbar spondylolisthesis. PI = 44°, PT = 14°, SS = 30°, LL = 28°, T5 to 12 = 9. CSL = Cobb angle of spondylolisthesis level, LL = lumbar lordosis, PI = pelvic incidence, PT = pelvic tilt, SS = sacral slope, SVA = sagittal vertical axis, T5 to T12 = Cobb from the fifth thoracic vertebral to 12th thoracic vertebral.

No significant differences in the age, gender, BMI, and T5 to T12 among 3 groups were noticed. The PI (73.1° ± 4.5°), SS (50.8° ± 3.9°), LL (53.1° ± 2.8°), SVA (66.1 ± 4.7 mm), PI–LL (20.0° ± 3.8°), and CSL (23.6° ± 2.2°) for TLDS were significantly larger than the PI (52.9° ± 4.8°), SS (32.2° ± 3.6°), LL (46.9° ± 3.5°), SVA (22.0 ± 8.0 mm), PI–LL (6.0° ± 1.3°), and CSL (20.0° ± 3.0°) for SLDS. Nevertheless, the PT/SS (44.0% ± 10.0%) for TLDS was significantly lower than PT/SS (63.8% ± 8.0%) for SLDS (Table 1, Fig. 1A and B). The PI (73.1° ± 4.5°), PT (22.3° ± 3.6°), SS (50.8° ± 3.9°), LL (53.1° ± 2.8°), PI–LL (20.0° ± 3.8°), and SVA (66.1 ± 4.7 mm) for TLDS were significantly larger than the PI (40.6° ± 5.0°), PT (17.1° ± 2.6°), SS (23.5° ± 3.6°), LL (32.5° ± 6.0°), PI–LL (8.1° ± 2.0°), and SVA (17.0 ± 8.0 mm) for normal group (NG). However, the PT/SS (44.0% ± 10.0%) for TLDS were significantly lower than PT/SS (74.0% ± 12.8%) for NG (Table 2, Fig. 1A and C). The PI (52.9° ± 4.8°), PT (21.0° ± 3.0°), SS (32.2° ± 3.6°), LL (46.9° ± 3.5°), and SVA (22.0 ± 8.0 mm) for SLDS were significantly higher than the PI (40.6° ± 5.0°), PT (17.1° ± 2.6°), SS (23.5° ± 3.6°), LL (32.5° ± 6.0°), and SVA (17.0 ± 8.0 mm) for NG. However, the PI–LL (6.0° ± 1.3°) and PT/SS (63.8% ± 8.0%) values for SLDS were significantly lower than PI–LL (8.1° ± 2.0°) and PT/SS (74.0% ± 12.8%) for NG (Table 3, Fig. 1B and C).

**Table 1:**
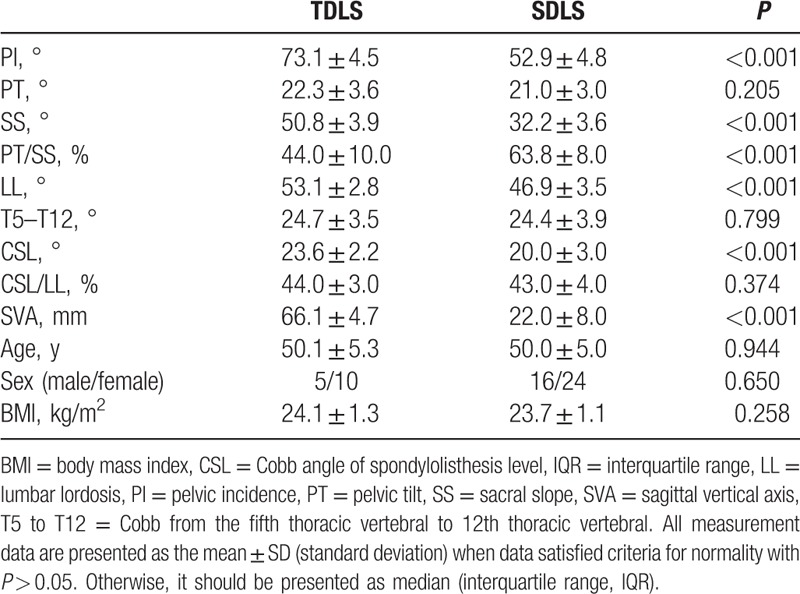
Comparison of spino-pelvic parameters between 2-level degenerative lumbar spondylolisthesis and single-level degenerative lumbar spondylolisthesis.

**Table 2:**
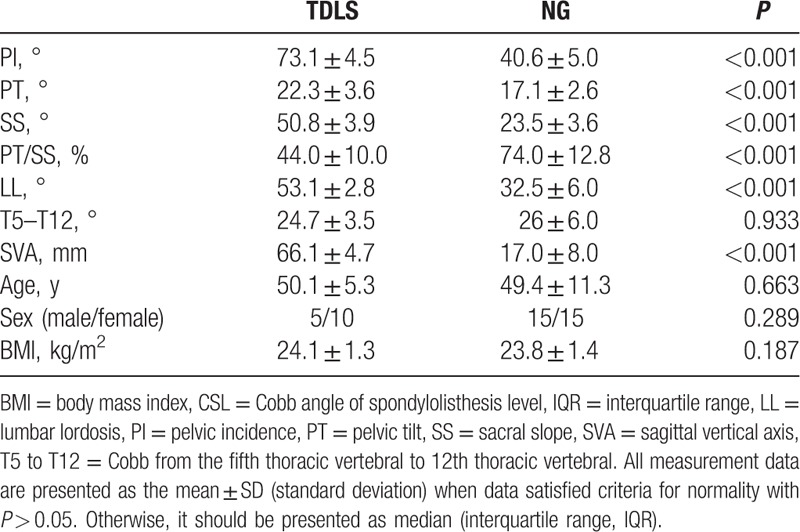
Comparison of spino-pelvic parameters between 2-level degenerative lumbar spondylolisthesis and normal group.

**Table 3:**
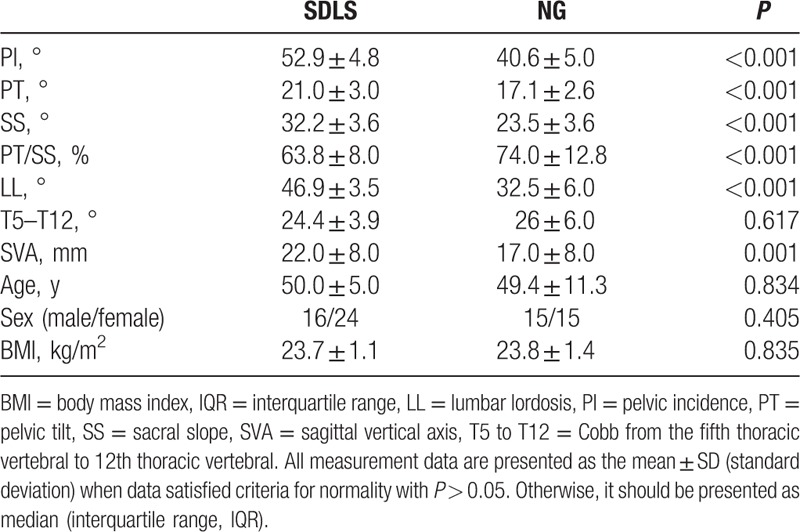
Comparison of spino-pelvic parameters between single-level degenerative lumbar spondylolisthesis and normal group.

**Figure 2:**
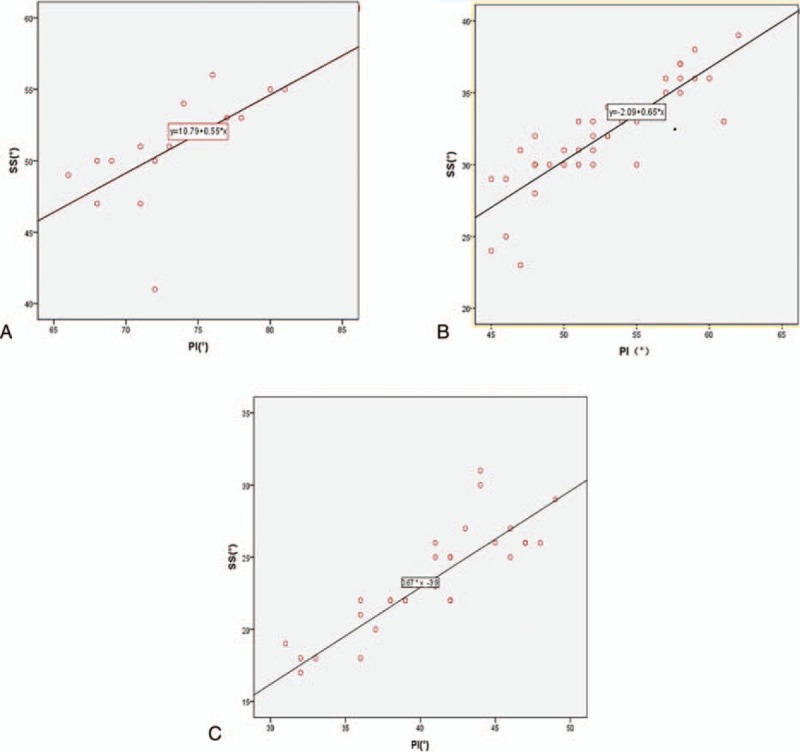
Correlation between pelvic incidence (PI) and sacral slope (SS) in among 2-level DS (TLDS) (A), single-level DS (SLDS) (B) and normal group (NG) (C). The line in TLDS is above that line in SLDS and the same tendency between SLDS and NG. However, the slope of line in TLDS is below the slope of line in SLDS, which suggests that the trend line of SS over PI in TLDS was downward when compared with SLDS and the same tendency between SLDS and NG.

**Figure 3:**
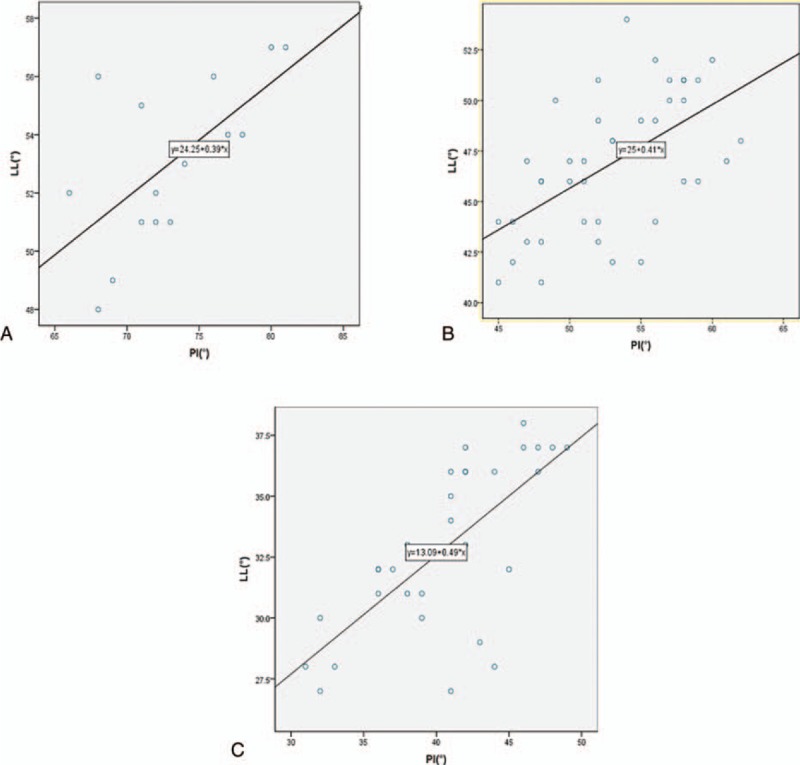
Correlation between pelvic incidence (PI) and lumbar lordosis in among 2-level DS (TLDS) (A), single-level DS (SLDS) (B), and normal group (NG) (C). The line in TLDS is above that line in SLDS and the same tendency between SLDS and NG. However, the slope of line in TLDS is below the slope of line in SLDS, which suggests that the trend line of sacral slope over PI in TLDS was downward when compared with SLDS and the same tendency between SLDS and NG.

With regard to the relationship between PI and positional parameters, SS and LL correlated well with PI in both TLDS and SLDS. The trend line of SS over PI (y = 10.79 + 0.55x, *r* = 0.408) and LL over PI (y = 0.39x + 24.25 *r* = 0.364) in TLDS were markedly upward as compared to SLDS (y = 0.65x − 2.09 *r* = 0.788; y = 0.41x + 25 *r* = 0.671, respectively); however, the slopes of equation of TLDS in SS over PI and LL over PI were smaller, compared to SLDS (Figs. 2A, 2B, 3A, 3B). Regarding the relationship between PI and positional parameters, SS and LL correlated well with PI in both TLDS and NG. The trend line of SS over PI (y = 10.79 + 0.55x, *r* = 0.408) and LL over PI (y = 0.39x + 24.25 *r* = 0.364) in TLDS were markedly upward as compared to NG (y = 0.67x − 3.9 *r* = 0.512; *y* = 0.49x + 13.09 *r* = 0.498, respectively); however, the slopes of equation of TLDS in SS over PI and LL over PI were smaller, compared to NG (Figs. 2A, 2C, 3A, 3C). Regarding the relationship between PI and positional parameters, SS and LL correlated well with PI in both SLDS and NG. The trend line of SS over PI (y = 0.65x − 2.09 *r* = 0.788) and LL over PI (y = 0.41x + 25 *r* = 0.671) in SLDS were markedly upward as compared to NG (y = 0.67x − 3.9 *r* = 0.512; *y* = 0.49x + 13.09 *r* = 0.498, respectively); however, the slopes of equation of SLDS in SS over PI and LL over PI were smaller, compared to NG (Figs. 2B, 2C, 3B, 3C).

## 4 Discussion

Spinopelvic sagittal alignment accompanied with biomechanical changes has been demonstrated in previous studies in the pathogenesis and development of lumbar degenerative diseases.^[22–24]^ Lim and Kim^[25]^ analyzed the differences of spinopelvic parameters between DS and IS and found that both DS and IS patients had a greater PI. He also reported that DS populations were likely to have higher SVA, compared to IS populations. Roussouly et al^[26]^ noted 2 different subgroups of sacro-pelvic balance for low-grade spondylolisthesis: the shear type (patients with high PI and SS have increased shear stresses at the lumbo-sacral junction, causing more tension on the pars interarticularis at L5); the nutcracker type (patients with a low PI and a smaller SS would have impingement of the posterior elements of L5 between L4 and S1 during extension. Based on K-means cluster analysis, Labelle et al^[27]^ and Mac-Thiong et al^[28]^ supported Roussouly opinion. To our knowledge, few studies focused on the spino-pelvic parameters for TLDS. The purpose of this study is to explore the sagittal spino-pelvic parameters for TLDS and compare between TLDS and SLDS in sagittal parameters. In addition, we try to explore the etiology of TLDS.

In our study, TLDS have higher PI, SS, SVA, PI–LL, and CSL than those in SLDS and the similar tend between SLDS and NG, PI, PT, SS, LL, PI–LL, and SVA are higher in SLDS groups. The trend lines of SS over PI and LL over PI in TLDS were markedly upward as compared to SLDS; however, the slopes of equation of TLDS in SS over PI and LL over PI were smaller, compared to SLDS. The same tendency occurred between SLDS and NG.

We analyzed between TLDS and SLDS according to difference of spondylolisthesis level and discovered that patients with TLDS had significantly larger PI (73.1°), SS (50.8°), LL (53.1°), PI–LL (20.0°), CSL (23.6°), and SVA (66.1 mm) than PI (52.9°), SS (32.2°), LL (46.9°), PI–LL (6.0°), CSL (20.0°), and SVA (22.0 mm) in SLDS. According to Ferrero,^[30]^ when PT ≥ 22°, SVA ≥ 47 mm, and PI–LL ≥ 11°, we regard spine as sagittal imbalance. Obviously, in our study, TLDS have a severe sagittal imbalance, but SLDS have not any. Initially, the shape of the pelvis, characterized by PI, processes retroversion. But as a compensatory mechanism, high SS and low LL would prevent sagittal imbalance. As time goes on, due to loss of compensatory mechanism, patients with high PI would have high SS and high LL, which generates a large amount of force on posterior facet joints, causing these mechanical stresses on posterior facets and accelerating facet arthrosis. The posterior facets arthrosis is associated with a significant inclination of the SS predispose slipping. This high lordosis induces a significant anterior displacement of the C7 plumbline and center of gravity. Serious loss of compensatory has high PI leading to TLDS. The factors, mentioned above, caused sagittal imbalance for patients with TLDS. Hence, the surgical treatment for TLDS is different from that for SLDS. As for SLDS, we select operative method to solve regional problem, as decompressed never root and bone fusion, but for TLDS, globe spine sagittal alignment should be considered in surgical choice.

Mac-Thiong et al^[28]^ indicated the position of the pelvis by the ratio of PT to SS (PT/SS). Our data showed significantly lower PT/SS in patients with TLDS than that in SLDS (43.0% and 68.3%, respectively) and lower PT/SS in SLDS compared to NG (68.3% and 74%, respectively), suggesting that a greater pelvic retroversion was with TLDS. As we know, a regional sagittal imbalance of the index segment is first compensated by adjacent mobile segments and then second by the pelvic orientation if the former compensation becomes insufficient.^[24,29]^ With SLDS, a significant extension of these segments should be alerted due to loss of compensation. TLDS has 2 level of spondylolisthesis, so it has more serious loss of compensation, causing the deeper the pelvis forward with larger PI, SS, PT, PI–LL, and SVA, implying that TLDS have a sagittal imbalance.

We found that patients with TLDS have a sagittal imbalance; the initial factors leading to these are worth studying. Ferrero et al^[30]^ and Lim and Kim^[25]^ have proven that high PI was the reason for DS. In addition, Vialle^[31]^ reported that a high PI is a predisposing factor of lumbar spondylolisthesis because PI is the arithmetic summation of SS and PT, meaning that high PI will necessarily predispose to a high SS and/or PT, and a high SS predisposes to a high LL in an attempt of the trunk to compensate and maintain the trunk centered over the femoral heads. We supported theirs. In addition, we observed the changing tendency of SS over PI and LL over PI among 3 groups and found that the relationship between PI and LL has more severe mismatches in TLDS, which suggested that in addition to high PI, other factors play significant roles in increasing the value of SS and LL in the patients with TLDS. The LL will structurally increase as a result of SS that gradually increases. Above all, TLDS was caused by multiple factors, and PI was a predicted factor.

The present study has several limitations. First, relatively low incidence of TLDS leads to a small sample size; second, this was just a retrospective study, and we also need a prospective study to explore the change progress of spondylolisthesis from the beginning; third, these data just form single center, and we need multicenter data in further study. However, to the best of our knowledge, this is the first report to explore sagittal spino-pelvic parameters for SLDS and compare it with TLDS and NG.

In conclusion, patients with TLDS had different sagittal alignments, compared to SLDS. Our studies showed that TLDS with higher PI, SVA, and PI–LL have severe sagittal imbalance, but SLDS have not any. So, the surgical selection for TLDS and SLDS is different. In order to improve clinical and functional outcome, correct and restore global sagittal alignment must be considered in treatment for TLDS. TLDS was caused by multiple factors, and PI was a predicted factor.

## References

[1] Weinstein JN Lurie JD Tosteson TD Surgical versus nonsurgical treatment for lumbar degenerative spondylolisthesis. N Engl J Med 2007;356:2257–70. 17538085 10.1056/NEJMoa070302 PMC2553804

[2] Smorgick Y Park DK Baker KC Single versus multilevel fusion for single-level degenerative spondylolisthesis and multilevel lumbar stenosis: four-year results of the spine patient outcomes research trial. Spine (Phila Pa 1976) 2013;38:797–805. 23169068 10.1097/BRS.0b013e31827db30f PMC3757550

[3] Schuller S Charles YP Steib JP Sagittal spinopelvic alignment and body mass index in patients with degenerative spondylolisthesis. Eur Spine J 2010;20:713–9. 21116661 10.1007/s00586-010-1640-2 PMC3082684

[4] Garet M Reiman MP Mathers J Nonoperative treatment in lumbar spondylolysis and spondylolisthesis: a systematic review. Sports Health 2013;5:225–32. 24427393 10.1177/1941738113480936 PMC3658408

[5] Fujiwara A Tamai K Yamato M The relationship between facet joint osteoarthritis and disc degeneration of the lumbar spine: an MRI study. Eur Spine J 1999;8:396–401. 10552323 10.1007/s005860050193 PMC3611192

[6] Devine JG Schenk-Kisser JM Skelly AC Risk factors for degenerative spondylolisthesis: a systematic review. Evid Based Spine Care J 2012;3:25–34. 23230415 10.1055/s-0031-1298615 PMC3516463

[7] Mariconda M Galasso O Imbimbo L Relationship between alterations of the lumbar spine, visualized with magnetic resonance imaging, and occupational variables. Eur Spine J 2007;16:255–66. 10.1007/s00586-005-0036-1 PMC2200682 16835739

[8] Berlemann U Jeszenszky DJ Buhler DW The role of lumbar lordosis, vertebral end-plate inclination, disc height, and facet orientation in degenerative spondylolisthesis. J Spinal Disord 1999;12:68–73. 10078953

[9] Berlemann U Jeszenszky DJ Buhler DW Mechanisms of retrolisthesis in the lower lumbar spine: a radiographic study. Acta Orthop Belg 1999;65:472–7. 10675942

[10] Boden SD Riew KD Yamaguchi K Orientation of the lumbar facet joints: association with degenerative disc disease. J Bone Joint Surg Am 1996;78:403–11. 8613448 10.2106/00004623-199603000-00012

[11] Duval-Beaupère G Schmidt C Cosson P A barycentremetric study of the sagittal shape of spine and pelvis: the conditions required for an economic standing position. Ann Biomed Eng 1992;20:451–62. 1510296 10.1007/BF02368136

[12] Legaye J Duval-Beaupère G Hecquet J Pelvic incidence: a fundamental pelvic parameter for three-dimensional regulation of spinal sagittal curves. Eur Spine J 1998;7:99–103. 9629932 10.1007/s005860050038 PMC3611230

[13] Aono K Kobayashi T Jimbo S Radiographic analysis of newly developed degenerative spondylolisthesis in a mean twelve-year prospective study. Spine (Phila Pa 1976) 2010;35:887–91. 20354469 10.1097/BRS.0b013e3181cdd1aa

[14] Barrey C Jund J Noseda O Sagittal balance of the pelvis-spine complex and lumbar degenerative diseases. A comparative study about 85 cases. Eur Spine J 2007;16:1459–67. 17211522 10.1007/s00586-006-0294-6 PMC2200735

[15] Rajnics P Templier A Skalli W The importance of spinopelvic parameters in patients with lumbar disc lesions. Int Orthop 2002;26:104–8. 12078871 10.1007/s00264-001-0317-1 PMC3620862

[16] Endo K Suzuki H Tanaka H Sagittal spinal alignment in patients with lumbar disc herniation. Eur Spine J 2010;19:435–8. 20091188 10.1007/s00586-009-1240-1 PMC2899756

[17] Barrey C Jund J Perrin G Spinopelvic alignment of patients with degenerative spondylolisthesis. Neurosurgery 2007;61:981–6. 18091275 10.1227/01.neu.0000303194.02921.30

[18] Lazennec JY Ramare S Arafati N Sagittal alignment in lumbosacral fusion relations between radiological parameters and pain. Eur Spine J 2000;9:47–55. 10766077 10.1007/s005860050008 PMC3611353

[19] Sanderson PL Fraser RD The influence of pregnancy on the development of degenerative spondylolisthesis. J Bone Joint Surg Br 1996;78-B:951–4. 10.1302/0301-620x78b6.1291 8951013

[20] Matsunaga S Sakou T Morizono Y Natural history of degenerative spondylolisthesis: pathogenesis and natural course of the slippage. Spine 1990;15:1204–10. 2267617 10.1097/00007632-199011010-00021

[21] Sato K Wakamatsu E Yoshizumi A The configuration of the laminas and facet joints in degenerative spondylolisthesis. Spine 1989;14:1265–71. 2603062 10.1097/00007632-198911000-00022

[22] Funao H Tsuji T Hosogane N Comparative study of spinopelvic sagittal alignment between patients with and without degenerative spondylolisthesis. Eur Spine J 2012;21:2181–7. 22639298 10.1007/s00586-012-2374-0 PMC3481103

[23] Aono K Kobayashi T Jimbo S Radiographic analysis of newly developed degenerative spondylolisthesis in a mean twelve-year prospective study. Spine (Phila Pa 1976) 2010;35:887–91. 20354469 10.1097/BRS.0b013e3181cdd1aa

[24] Barrey C Jund J Noseda O Sagittal balance of the pelvis-spine complex and lumbar degenerative diseases. A comparative study about 85 cases. Eur Spine J 2007;16:1459–67. 17211522 10.1007/s00586-006-0294-6 PMC2200735

[25] Lim JK Kim SM Difference of sagittal spinopelvic alignments between degenerative spondylolisthesis and isthmic spondylolisthesis. J Korean Neurosurg Soc 2013;53:96–101. 23560173 10.3340/jkns.2013.53.2.96 PMC3611066

[26] Roussouly P Gollogly S Berthonnaud E Sagittal alignment of the spine and pelvis in the presence of L5-S1 isthmic lysis and low-grade spondylolisthesis. Spine 2006;31:2484–90. 17023859 10.1097/01.brs.0000239155.37261.69

[27] Labelle H Roussouly P Berthonnaud E Mac-Thiong JM Hresko T Dimar J Parent S Weidenbaum M Brown C Hu S Spondylolisthesis classification based on spino-pelvic alignment, Podium presentation at the 2009 Scoliosis Research Society Annual Meeting, San Antonio, USA.

[28] Mac-Thiong JM Roussouly P Hresko MT Labelle H The importance of sagittal spino-pelvic alignment in low grade spondylolisthesis. Identification of subgroups based on Pelvic Incidence and Sacral Slope, Podium presentation at the Scoliosis Research Society Annual Meeting, Kyoto, Japan, September 2010.

[29] Le Huec JC Charosky S Barrey C Sagittal imbalance cascade for simple degenerative spine and consequences: algorithm of decision for appropriate treatment. Eur Spine J 2011;20 (suppl 5):699–703. 21811823 10.1007/s00586-011-1938-8 PMC3175932

[30] Ferrero E Ould-Slimane M Gille O French Spine Society (SFCR) Sagittal spinopelvic alignment in 654 degenerative spondylolisthesis. Eur Spine J 2015;24:1219–27. 25652553 10.1007/s00586-015-3778-4

[31] Vialle R Ilharreborde B Dauzac C Is there a sagittal imbalance of the spine in isthmic spondylolisthesis? A correlation study. Eur Spine J 2007;16:1641–9. 17437136 10.1007/s00586-007-0348-4 PMC2078287

